# Cost of interventions to control schistosomiasis: A systematic review of the literature

**DOI:** 10.1371/journal.pntd.0008098

**Published:** 2020-03-30

**Authors:** Paola Salari, Thomas Fürst, Stefanie Knopp, Jürg Utzinger, Fabrizio Tediosi

**Affiliations:** 1 Swiss Tropical and Public Health Institute, Basel, Switzerland; 2 University of Basel, Basel, Switzerland; Chinese Center for Disease Control and Prevention, CHINA

## Abstract

**Background:**

Schistosomiasis, a disease caused by blood flukes of the genus *Schistosoma*, belongs to the neglected tropical diseases. Left untreated, schistosomiasis can lead to severe health problems and even death. An estimated 800 million people are at risk of schistosomiasis and 250 million people are infected. The global strategy to control and eliminate schistosomiasis emphasizes large-scale preventive chemotherapy with praziquantel targeting school-age children. Other tools are available, such as information, education, and communication (IEC), improved access to water, sanitation, and hygiene (WASH), and snail control. Despite available evidence of the effectiveness of these control measures, analyses estimating the most cost-effective control or elimination strategies are scarce, inaccurate, and lack standardization. We systematically reviewed the literature on costs related to public health interventions against schistosomiasis to strengthen the current evidence-base.

**Methodology:**

In adherence to the PRISMA guidelines, we systematically searched three readily available electronic databases (i.e., PubMed, WHOLIS, and ISI Web of Science) from inception to April 2019 with no language restrictions. Relevant documents were screened, duplicates eliminated, specific rules on studies to consider were defined, and the eligible studies fully reviewed. Costs of schistosomiasis interventions were classified in three groups: (i) preventive chemotherapy; (ii) preventive chemotherapy plus an individual diagnostic test to identify at-risk population; and (iii) test-and-treat interventions.

**Principal findings:**

Fifteen articles met our inclusion criteria. In general, it was hard to compare the reported costs from the different studies due to different approaches used to estimate and classify the costs of the intervention assessed. Costs varied considerably from one study to another, ranging from US$ 0.06 to US$ 4.46 per person treated. The difference between financial and opportunity costs only played a minimal role in the explanation of the costs’ variation, even if delivery costs were two times higher in the analyses including economic costs. Most of the studies identified in our systematic review focused on sub-Saharan African countries.

**Conclusions/Significance:**

The degree of transparency of most of the costing studies of schistosomiasis interventions found in the current review was limited. Hence, there is a pressing need for strategies to improve the quality of cost analyses, and higher reporting standards and transparency that should be fostered by peer-review journal policies. Cost information on these interventions is crucial to inform resource allocation decisions and those regarding the affordability of scaling-up interventions.

## Author summary

Schistosomiasis is one of about 20 neglected tropical diseases. It affects millions of people mainly in poor settings in African countries. The global strategy to control and eliminate schistosomiasis emphasizes large-scale preventive chemotherapy with praziquantel targeting school-age children. Evidence of the effectiveness of these control measures is largely available, while less is known about the costs of their implementation. We systematically reviewed the literature and analyzed studies that estimated costs of schistosomiasis interventions. We summarized the available evidence in three intervention groups: (i) preventive chemotherapy; (ii) preventive chemotherapy plus an individual diagnostic test to identify at-risk population; and (iii) test-and-treat interventions. Costs were overall hard to compare and varied considerably from one study to another. There is a need for higher reporting standards and transparency that should be fostered by peer-review journal policies. Cost information on these interventions is crucial to inform resource allocation decisions and those regarding the affordability of scaling-up interventions.

## Introduction

Over the past 15 years, neglected tropical diseases (NTDs) have received increasing attention by the global health community. In 2012, for instance, the World Health Organization (WHO) published a roadmap for implementation with the aim to accelerate the control and elimination of NTDs by setting disease-specific targets [1]. In the meantime, several WHO reports provided updates on progress and remaining challenges of the control and elimination of NTDs [2–4]. Also in 2012, the London Declaration on NTDs was endorsed by a number of public and private organizations that formed the “Uniting to Combat Neglected Tropical Diseases” [5]. The London Declaration is committed to support the eradication of dracunculiasis; the elimination of blinding trachoma, human African trypanosomiasis, leprosy, and lymphatic filariasis; and the control of Chagas disease, onchocerciasis, schistosomiasis, soil-transmitted helminthiasis, and visceral leishmaniasis by 2020. Importantly, NTDs are also explicitly mentioned under target 3.3 of Sustainable Development Goal (SDG) 3.

Schistosomiasis is caused by blood flukes of the genus *Schistosoma*. Infection occurs when humans contact stagnant or slow-flowing freshwater bodies that are contaminated by infective parasite larvae that are released from specific freshwater snails. The larvae penetrate the unbroken human skin and develop within a couple of weeks into adult worms that pair up and start producing eggs. While some eggs are released in the urine or feces (depending on the *Schistosoma* species), other eggs are trapped in the tissue and cause an inflammatory reaction. Heavy infections that are left untreated can lead to anemia, stunted growth, severe damage of organs, and even death [6, 7]. There are an estimated 800 million people at risk of schistosomiasis [8]. Of the 250 million infected individuals, 200 million live in Africa. In 2017, the global burden of schistosomiasis was estimated at 1.43 million disability-adjusted life years (DALYs); the third highest burden among the NTDs [9].

Schistosomiasis can be prevented by avoiding contact with contaminated freshwater, and the risk of infection can be reduced through improved access to water, sanitation, and hygiene (WASH), and information, education, and communication (IEC) [10]. Risks of infection are related to occupational exposures, such as fishing in rivers and lakes, which are an important source of income for households in endemic areas [11], and recreational activities such as swimming, bathing, and playing in the water. Additionally, as Asian schistosomiasis (most important species is *Schistosoma japonicum*) is a zoonosis, preventive measures should be directed toward infections in animals as well, particularly water buffaloes, which act as an important reservoir for the parasite [12].

The current mainstay of schistosomiasis control is the periodic administration of praziquantel to at-risk groups (e.g., school-age children), an approach phrased preventive chemotherapy. While this strategy does not prevent infection or reinfection, it reduces morbidity and might also impact on transmission. For large-scale preventive chemotherapy, WHO recommends a single 40 mg/kg oral dose of praziquantel, with the frequency of drug administration depending on the prevalence of infection among school-age children [13]. In 2012, WHO set the targets to (i) control schistosomiasis by 2020; (ii) eliminate schistosomiasis as public health problem by 2025; and (iii) interrupt transmission in selected countries by 2025 [2]. At present, coverage of preventive chemotherapy among school-age children is the main process indicator, though more quantitative impact indicators are required, as has been proposed for soil-transmitted helminthiasis [14, 15].

The emphasis on preventive chemotherapy against schistosomiasis and several other NTDs is mainly explained through the available evidence of this strategy for morbidity control. However, analyses determining the most cost-effective control or elimination strategies in different settings are required, including accurate cost data of the interventions. Such information is critical to inform resource allocation decisions and affordability of scaling-up interventions. Yet, studies aimed at estimating resources used and related costs of schistosomiasis control interventions require substantial efforts in terms of collecting and analyzing appropriate data. Methodological guidelines on economic evaluations and cost studies of health interventions are available [16–17]. Recently, a global consortium on costing global health interventions was established and tasked to produce a “reference case for global health costing interventions” [18]. A recent systematic review of cost and cost-effectiveness studies of soil-transmitted helminthiasis control programs highlighted major gaps, such as the absence of cost data and inconsistencies in methodologies adopted to conduct these studies [19].

To our knowledge, there is no recent review of the evidence available regarding the costs of schistosomiasis interventions. Hence, the goal of this study was to fill this gap. We systematically reviewed the literature on costs and cost components related to public health interventions targeting schistosomiasis, adhering to the PRISMA guidelines [20].

## Methods

### Ethics statement

The data utilized in the current systematic review were published elsewhere. Hence, the interested reader is referred to the original publications from which the current data were extracted, with regard to details about ethics approval, informed consent procedures, and schistosomiasis control interventions.

### Literature search

**Fig 1:**
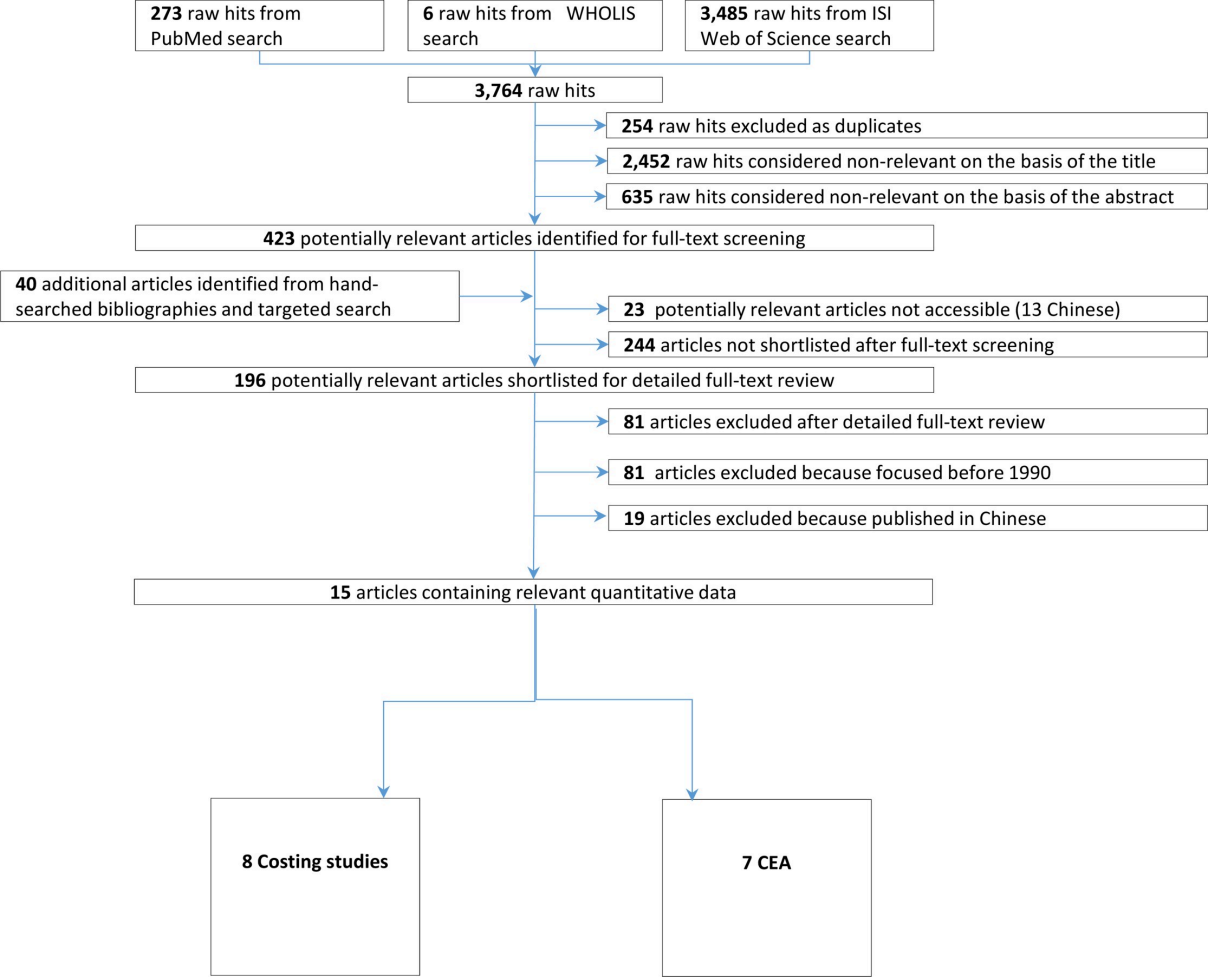
PRISMA flow diagram.

Our search strategy is summarized in Fig 1. Further details are provided in Supporting Information (S1 and S2 Tables). The keywords used were different combinations of “*Schistosoma*/schistosomiasis”, “economics/costs”, and “program evaluation”. Of note, no exclusion criteria were imposed at this step based on the date, location, or type of study or type of intervention. Similarly, in the first stage, no studies were excluded based on language or type of publication (e.g., peer-reviewed journals and reports from grey literature).

All information on cost were considered eligible at this stage, from costs actually incurred to implement a past or existing intervention to theoretical cost predictions from hypothetical intervention scenarios. However, simple cost claims solely reported in tentative budgets, non-modeling secondary sources, and opinion pieces (e.g., commentaries, editorials, or viewpoints) were excluded in order to avoid biased, invalid, unreliable, or misrepresented information. In these cases, efforts were made to explore whether the respective statements were based on relevant underlying primary data. If so, the original references containing the respective cost or cost evaluation were assessed.

Likewise, efforts were made to access the original references and primary data if mentioned in review articles and book chapters. The latter were only considered if the former were not accessible. References reporting only on cost of single material items (e.g., only purchasing prize of a drug or diagnostic filter paper) were excluded as interested readers can obtain this information most accurately directly from the manufacturer. Cost-of-illness information (e.g., cost due to schistosomiasis-related work productivity loss) were not considered as these are consequences of *Schistosoma* spp. infection and not cost of schistosomiasis control efforts, with the exception of treatment cost (e.g., case management of severe schistosomiasis patients), which has been included.

The bibliographies of the finally included documents were hand-searched for potential additional references. Furthermore, attempts were made to access data mentioned in the respective documents, but not yet identified through the described search.

S2 and S3 Tables in Supporting Information show the databases searched and respective specifications, thesauri, complete search terms with Boolean operators and wildcards (i.e., the asterisk), and the resulting number of raw hits. In particular, S3 Table shows the details of the search strategy with results summarized in S2 Table. Box 1 below provides a definition of the economic terms used in this manuscript.

**Box 1: Glossary**

Costs: Defined as the value of resources used to provide a service or an intervention that can be categorized as financial or economic costs.

Financial costs: Represent actual monetary transactions, i.e., all the costs generally considered in the accountancy of a project. In this category included are, among others, the salaries of people involved in the study, the transport costs such as the cost of gasoline, or the cost of the material and equipment.

Opportunity costs: Defined as the costs of the best alternatives that have been forgone by using a resource in that specific way (e.g., the time of volunteers).

Economic costs: In addition to actual financial expenditures on goods and services, they include a valuation of resources that do not have financial transactions (i.e., opportunity costs)–such as the time of volunteers, donated goods, services or capital goods, and inputs whose prices are distorted.

Perspective: The perspective of the study is defined as the viewpoint from which a cost analysis is conducted. The perspective indicates which costs have been included in the analysis. Provider perspective includes only costs incurred by the provider whether public (such as the Ministry of Health) or private, in the provision of an intervention or a service. The societal perspective includes, instead, not only the provider costs, but also out of pocket costs incurred by individuals and households (such as costs associated with travel to health facilities and waiting at service delivery points).

Secondary data: Data not collected specifically for the costing study, but already existing and collected by others (not by the authors of the analysis).

Hypothetical costs: These are costs that are imputed, derived from statistical models, or readapted from the literature.

### Organization and screening of literature

To identify all relevant documents, duplicates were removed. The remaining references were first screened by title and then by abstract. References still considered potentially relevant were further assessed in a two-stage process. First, the full-texts were screened for reporting any quantitative monetary values. Second, the shortlisted references underwent detailed full-text review to decide on final inclusion or exclusion. The detailed full-text review was completed by two reviewers (PS and TF). The whole process is illustrated in Fig 1, a PRISMA flowchart [20].

In parts, the data analysis is inspired by a recently published systematic review on the cost of interventions targeting soil-transmitted helminth infection [19] and also by the Bill & Melinda Gates Foundation reference case for economic evaluations [16]. Initially, all included references were classified based on three characteristics. First, the type of economic analysis was considered. References that mainly reported on costs of interventions were classified as costing analyses in line with current standard textbook definitions [21, 22]. Second, references that were partly or fully based on hypothetical cost, budget, and/or intervention data were separated from references that were purely based on truly incurred costs and really implemented interventions. Third, the level of detail of the reported cost information was evaluated. The references were classified as only reporting lump sum costs if they provided only a single overall cost figure. If the costs were still largely aggregated, but at least split up in a few major cost categories, the respective reference was considered as macro-level cost study. If detailed, itemized costs were reported and the respective reference was considered as micro-level cost study.

After these broad classification steps, specific decisions were made on which papers to keep in the final version of the selection process. Articles describing studies and costs done before 1990 were excluded. Comparing cost estimates from studies conducted three or more decades ago with more recent cost estimates is in fact challenging due to changes in both interventions modalities, socioeconomic, and health sector context. Additionally, most of the articles reporting cost estimates prior to 1990 did not meet other inclusion criteria. Lastly, 19 studies published in Chinese were excluded from the analysis in the last selection step. Among these Chinese articles, 10 did not include cost estimates, while in the remaining 9 studies the costs included were either only aggregated or it could not be deciphered how exactly the estimates had been generated.

The studies that reported costs only as lump-sum and without further disaggregation were excluded for two reasons. First, the aim of our study was to compare not only the total cost, but also the estimates of different cost categories (e.g., personnel and transport). Second, for most of the studies, it was impossible to understand the exact methodology used, and hence, the reliability of the cost estimates was questionable. Finally, costing studies based on hypothetical costs were also excluded. Taken together, only costs based on primary or secondary data that were identifiable were included.

### Data extraction

Tables 1 and 2 show the selected studies. Each row of the tables corresponds to one cost estimate. The articles including more than one analysis are reported in different lines. Tables 1 and 2 also summarize the most important characteristics for each cost analysis included.

**Table 1:** List of costs analyses of group A (i.e., preventive chemotherapy with or without an educational component) and their main characteristics.

Reference	Group of studies per intervention	Country	Description of the intervention/treatment	Target of intervention	Type of economic analysis	Economic perspective explicitly stated	Economic costs included (Y/N)
Brooker et al. (2008)	A	UGANDA	Nationwide school-based MDA. Mass treatment with praziquantel to treat schistosomiasis and with albendazole to treat soil-transmitted helminths was given to all schools and communities in targeted areas	Schistosomiasis and STH	CEA	Government	Y
Evans et al. (2011)	A	NIGERIA	Annual MDA with ivermectin (for onchocerciasis), albendazole (for STH and with ivermectin for LF) and praziquantel (for schistosomiasis) **Standard alone (SA)**	Schistosomiasis onchocerciasis, LF and STH	CEA	NA	N
Evans et al. (2011)	A	NIGERIA	Annual MDA with ivermectin (for onchocerciasis), albendazole (for STH and with ivermectin for LF) and praziquantel (for schistosomiasis). **Triple drug administration (TDA)**	Schistosomiasis onchocerciasis, LF and STH	CEA	NA	N
Gabrielli et al. (2006)	A	BURKINA FASO	MDA on the entire school-age population of Burkina Faso with praziquantel against schistosomiasis and albendazole against STH	Schistosomiasis	Costing	NA	Y
Guo et al. (2005)	A	CHINA	MDA	Schistosomiasis	Costing	NA	N
Guyatt et al. (1994)	A	TANZANIA	Annual MDA of all primary school-children by a mobile team comprising one driver, one fieldworker and one Rural Medical Aid. All present children of 77 schools were treated with a single oral dose of praziquantel	Schistosomiasis	CEA	Health care's provider	N
Guyatt et al. (1994)	A	TANZANIA	Annual MDA of all primary school-children by a mobile team comprising one driver, one fieldworker and one Rural Medical Aid. All present children of 77 schools were treated with a single oral dose of praziquantel	Schistosomiasis	CEA	Health care's provider	Y
Kabatereine et al. (2006)	A	UGANDA	National control program	Schistosomiasis and STH	Costing	NA	N
Leslie et al. (2011)	A	NIGER	The study examines the economic costs of the **MDA vertical program** in its first and second years	Schistosomiasis and STH	CEA	NA	Y
Leslie et al. (2013)	A	NIGER	Costs of **integrated program** for LF, schistosomiasis, trachoma and soil-transmitted helminthiasis, done in 2009. Treatment was provided to schoolchildren by teachers in primary schools. Adults and other children not in school received treatment from community distributers and health workers in the village	Schistosomiasis, LF, trachoma and STH	Costing	NA	Y
Linehan et al. (2011)	A	BURKINA FASO, GHANA, MALI, NIGER, UGANDA, SIERRA LEONE, HAITI (only LF and STH in HAITI)	**Integrated NTD control programs**. Disease-specific mapping was carried out. Diagnostic approaches for mapping: 1) detecting eggs in urine or stool (microscopy). 2) detecting blood in urine (hemastix or questionnaires)	Schistosomiasis, onchocerciasis, LF, STH, trachoma	Costing	NA	N
Oshish et al. (2011)	A	YEMEN	In preparation for a 6-year nationwide control program with the aim of expanding treatment to the wider community, a new programmatic approach of complementing school-based distribution with community-based treatment was trialed in 10 highly endemic districts	Schistosomiasis and STH	Costing	NA	N
Talaat & Evans (2000)	A	EGYPT	The school-based health program for schistosomiasis control adopted by the Egyptian Ministry of Health and Population focuses on treating enrolled schoolchildren	Schistosomiasis	CEA	NA	Y
Yu et al. (2002)	A	CHINA	MDA to all the villagers except those not able to take praziquantel	Schistosomiasis	CEA	Health care's provider	N

Notes: In column “Target of intervention” STH stands for soil-transmitted helminths and LF for lymphatic filariasis. In column “Type of economic analysis” CEA indicates a cost-effectiveness analysis.

**Table 2:** List of costs analyses of group B (i.e., preventive chemotherapy plus an individual test to identify at-risk population) and group C (test-and-treat interventions) and their main characteristics.

Reference	Group of studies per intervention	Country	Description of the intervention/treatment	Reference year(s) for intervention (s) and cost(s)	Target of intervention	Type of economic analysis	Economic perspective explicitly stated	Economic costs included (Y/N)
Croce et al. (2010)	B	CAMBODIA	The program was based on a MDA carried out by Center for Malaria Control staff, who reached the villages using boats and local volunteers. An average of 2,000 stool samples from individuals in randomly selected villages were collected for parasitologic survey and analyzed with the Kato-Katz method	1995–2006	Schistosomiasis	CEA	Ministry of Health	Y
Partnership for Child Development (1998) Health Policy Plan	B	TANZANIA	The intervention has been done after giving a questionnaire to students to estimate the prevalence in schools of schistosomiasis. The schools in which the prevalence of reported schistosomiasis was 25% were selected for MDA with praziquantel	1996	Schistosomiasis	Costing	NA	Y
Partnership for Child Development (1998)	B	TANZANIA	The intervention has been done after giving a questionnaire to students to estimate the prevalence in schools of schistosomiasis. The schools in which the prevalence of reported schistosomiasis was 25% were selected for MDA with praziquantel	1996	Schistosomiasis	Costing	NA	Y
Partnership for Child Development (1999)	B	GHANA	**Selective mass treatment, financial costs**. MDA was given in all schools in which 30% of children said they had blood in urine, while selective treatment was given in the remaining schools to all children who reported blood in their urine. A questionnaire was used to identify schools where MDA was required for urogenital schistosomiasis	1996	Schistosomiasis	Costing	NA	Y
Partnership for Child Development (1999)	B	GHANA	**Selective mass treatment, economic costs**	1996	Schistosomiasis	Costing	NA	Y
Guo et al. (2005)	C	CHINA	Two highly endemic villages were selected to compare the strategy of ‘passive chemotherapy’ plus health education to that of MDA singly. Under ‘passive chemotherapy’ they mean a concept whereby medical teams treat residents in schistosome-endemic areas with praziquantel upon their request	1998–2000	Schistosomiasis	Costing	NA	N
Guyatt et al. (1994)	C	TANZANIA	Teachers annually screened children using Sangur reagent strips and referred all positives to the nearest dispensary for treatment. **Financial costs**	1991	Schistosomiasis	CEA	Health care's provider	N
Guyatt et al. (1994)	C	TANZANIA	Teachers annually screened children using Sangur reagent strips and referred all positives to the nearest dispensary for treatment. **Economic costs**	1991	Schistosomiasis	CEA	Health care's provider	Y
Guyatt et al. (1994)	C	TANZANIA	Control was provided by passive case detecting using urine sedimentation and subsequent treatment of positives with a single oral dose of praziquantel (40 mg/kg). **Financial costs**	1991	Schistosomiasis	CEA	Health care's provider	N
Guyatt et al. (1994)	C	TANZANIA	Control was provided by passive case detecting using urine sedimentation and subsequent treatment of positives with a single oral dose of praziquantel (40 mg/kg). **Economic costs**	1991	Schistosomiasis	CEA	Health care's provider	Y
Talaat & Evans (2000)	C	EGYPT	The school-based health program for schistosomiasis control adopted by the Egyptian Ministry of Health and Population focused on treating enrolled schoolchildren. Screening involved only urine using the simple sedimentation technique. Selective chemotherapy for out-of-school children	1999	Schistosomiasis	CEA	NA	Y
Yu et al. (2002)	C	CHINA	**Clue** chemotherapy, consisting of treatment to those with contact with infected water and/or symptoms of infection. Costs reported here are those for years 1 and 2. The paper also shows the total costs divided by year and by activity (training, supervision, mobilization, diagnosis, and treatment).	1998–2000	Schistosomiasis	CEA	Health care's provider	N
Yu et al. (2002)	C	CHINA	**Screen** chemotherapy-treatment prescribed to the stool egg positive cases after Kato-Katz examination	1998–2002	Schistosomiasis	CEA	Health care's provider	N

Notes: In column “Type of economic analysis” CEA indicates a cost-effectiveness analysis.

In addition to the three characteristics that we used to select the studies in the previous phase–type of economic analysis (cost or cost-effectiveness analysis study), level of detail of costing data (macro *versus* micro), and cost data type (primary, secondary, hypothetical, or mix)–the final classification of the costs included also other essential features. First, we identified the type of intervention: (i) preventive chemotherapy; (ii) test-and-treat interventions where the at-risk population was identified through disease mapping or questionnaire assessment, diagnostic test plus individual treatment; and (iii) behavioral control or education interventions. Second, we checked the stage of intervention, i.e., if it was routinely run or newly starting. Finally, we also tabulated the type of costs included (financial or economic) and the economic perspective adopted, where explicitly stated. Of note, our data analysis is based mainly on the type of intervention.

### Data analysis

The costs of each study were analyzed in several steps. First, we assessed the total cost and the cost structure (i.e., how the total cost is disaggregated into categories) reported by each study. For the sake of cost comparison, we tried to adapt each cost category into standardized ones, also grouping some of them together, to obtain uniform and comparable cost categories for each study. To this end, the final standardized cost categories are “personnel and training”, “human drugs”, “materials and equipment”, “costs for running the program, transport, and management”, “diagnostic test” (e.g., questionnaire), and “behavior change”. The costs for each intervention and for each category were reported as unit costs (Table 3). They were computed in terms of people treated [23–30], treatments delivered [31–33], people targeted [35, 36], people surveyed [30], or people protected [37].

**Table 3:** List of analyses and their unit costs, ordered by groups of interventions.

Reference	Group of studies per intervention	Total cost (US$) inflated to 2018	Costs for personnel and training	Cost for human drugs	Costs for materials and equipment	Costs for running the program, transport and management	Cost for diagnostic test	Cost for behavior change	Other costs	Units of measurement used
Evans et al. (2011)	A	0.054	0.472	-	0.372	0.275	-	-	-	Treatments delivered
Kabatereine et al. (2006)	A	0.058	0.123	0.513	-	0.486	-	0.457	-	People targeted
Evans et al. (2011)	A	0.091	0.839	-	0.198	0.544	-	-	-	Treatments delivered
Linehan et al. (2011)	A	0.161	-	0.365	-	0.774	0.177	0.355	-	Treatments delivered
Gabrielli et al. (2006)	A	0.408	0.555	0.283	0.737	0.512	-	0.155	-	Children treated
Oshish et al. (2011)	A	0.647	0.730	0.292	-	0.196	-	0.545	0.336	People targeted
Leslie et al. (2011)	A	0.689	-	0.335	0.154	0.165	-	-	0.173	Treatments delivered
Brooker et al. (2008)	A	0.689	0.116	0.272	-	0.179	-	0.122	-	People treated
Guo et al. (2005)	A	0.827	0.653	0.762	-	-	-	-	-	People treated
Guyatt et al. (1994)	A	1.445	0.166	1.263	-	0.665	-	-	0.592	People treated
Guyatt et al. (1994)	A	1.450	0.173	1.219	-	0.668	-	-	0.142	People treated
Yu et al. (2002)	A	1.839	1.247	0.321	0.468	0.276	-	-	0.131	People surveyed
Talaat & Evans (2000)	A	2.281	0.560	1.193	0.882	0.450	-	-	0.000	Children treated
Leslie et al. (2013)	A	4.461	0.543	4.245	0.196	0.320	-	-	0.117	Treatments delivered
Partnership for Child Development (1998)	B	1.195	0.131	0.876	-	0.786	0.875	-	0.278	Children treated
Croce et al. (2010)	B	1.265	-	0.219	-	0.897	0.128	-	0.211	People protected
Partnership for Child Development (1999)	B	1.848	0.322	0.835	-	0.649	0.295	-	0.729	Children treated
Partnership for Child Development (1998)	B	1.996	0.131	0.876	-	0.574	0.388	-	0.278	Children treated
Partnership for Child Development (1999)	B	4.452	0.337	0.876	-	1.673	1.957	-	0.713	Children treated
Guo et al. (2005)	C	0.349	0.571	0.233	0.582	-	-	-	0.000	People treated
Guyatt et al. (1994)	C	0.812	0.689	0.259	0.449	0.349	-	-	0.244	People treated
Guyatt et al. (1994)	C	0.837	0.879	0.259	0.454	0.360	-	-	0.822	People treated
Yu et al. (2002)	C	1.215	0.792	0.397	0.258	-	-	-	0.000	People surveyed
Yu et al. (2002)	C	1.320	0.776	0.528	0.168	-	-	-	0.697	People treated
Guyatt et al. (1994)	C	1.965	0.570	1.142	0.613	0.552	-	-	0.985	People treated
Guyatt et al. (1994)	C	2.069	0.163	1.219	0.645	0.579	-	-	0.232	People treated
Talaat & Evans (2000)	C	2.513	0.552	0.774	0.753	0.435	-	-	-	Children screened

For some studies, it was hard to compute the unit cost because the output was not explicitly stated. For example, one article was excluded in the final list [38], because it did not clearly mention the number of people treated and it was not possible to compare it with the others. Another challenge arose during the analyses, because interventions not only targeted schistosomiasis but instead also other NTDs, such as lymphatic filariasis, onchocerciasis, trachoma, and soil-transmitted helminthiasis. For these analyses, it was not always possible to disentangle the cost of the intervention against schistosomiasis from the other interventions. Hence, the unit cost was considered as an average cost of all the interventions mentioned. All costs in the identified studies were presented in US$. We inflated the costs to 2018 US$, using the Consumer Price Index (CPI) inflator rate (http://www.inflation.eu/inflation-rates/united-states/historic-inflation/cpi-inflation-united-states.aspx) [39].

To enhance comparability of results, we divided the studies by type of intervention, in three groups. Group A included 14 cost estimates where preventive chemotherapy intervention was implemented with [23, 26, 33, 34, 36] or without [27, 28, 30–32, 35] a behavioral change or education interventions. One of the analyses included also non-schistosomiasis interventions [32], one included a non-schistosomiasis intervention plus an individual diagnostic test [33], and one included the identification of at-risk population [34].

In Group B, only five cost estimates were included [24, 25, 37]. They all estimated preventive chemotherapy interventions accompanied by a method of identification of at-risk population. One cost analysis [37] included identification of at-risk population (annual surveys were also conducted in primary schools) and an individual diagnostic test (Kato-Katz thick smear method for examination of stool samples).

Group C was most heterogeneous and did not include preventive chemotherapy interventions, but only treatments of identified populations at risk [27–30]. In one study [28], for example, teachers annually screened children using reagent strips for microhematuria (a proxy for *S*. *haematobium* infection) and referred all positives to the nearest dispensary for treatment. In another study [30] the authors compared a “clue chemotherapy”, consisting of treatment of those with contact with infected water and/or symptoms of infection, with a “screen chemotherapy”, where treatment was prescribed to the stool egg-positive cases after Kato-Katz thick smear examination. In another analysis [29], the authors employed a screening method, which involved only urine examination, using a sedimentation technique. All the analyses included in group C were presented in the original papers as additional (and not main) analyses, and compared with the main cost analysis. The latter is presented in groups A or B and is focused on preventive chemotherapy.

## Results

At the end of this process, 15 articles corresponding to our selection criteria were identified. Most of the included manuscripts reported several cost estimates. In total, there were 27 cost estimates. This selection included 19 estimates of preventive chemotherapy costs and 8 of treatments preceded by a diagnostic test. None of the retained studies estimated the costs of snail control interventions, while six studies included a behavioral control intervention.

Most of the studies identified through our systematic review focused on sub-Saharan African countries, namely Burkina Faso [26, 34], Ghana [25, 34], Mali [34], Nigeria [31], Niger [32–34], Sierra Leone [34], Tanzania [24, 28] and Uganda [23, 34, 35]. Some studies were from North Africa (e.g., Egypt [29]) and the Middle East (e.g., Yemen, [36]), and from Asia, with the People’s Republic of China [27, 30] and Cambodia [37] both being represented. Finally, one study included estimates referred to Haiti [34] beside the aforementioned African countries; yet, only soil-transmitted helminths and lymphatic filariasis were treated in Haiti, as schistosomiasis is not endemic.

### Study perspective

The economic perspective of the studies included in our review varied. In nine analyses, the authors clearly stated that the health care provider’s perspective was adopted. In one analysis, the Ministry of Health’s perspective was considered, while in another study, the perspective of the Government was employed. The remaining 16 cost analyses did not specify any perspective. Yet, implicitly, these studies adopted a control program logic. Unexpectedly, the type of economic perspective did not correlate with higher or lower costs. This is probably due to the fact that, despite the declared perspective differed among the final set of analyses, most studies included the same types of costs. It may indicate that most studies did not carefully consider the perspective of the analyses, which would be consistent to the findings of other reviews pertaining to interventions targeting NTDs, such as a recent review focussing on soil-transmitted helminthiasis control programs [19].

### Comparison of total costs

As shown in Table 3, costs varied considerably among studies and analyses. The minimum cost per person treated was US$ 0.06 [35], while the maximum was US$ 4.46 [28]. Twelve analyses reported a cost below US$ 1; ten analyses between US$ 1 and US$ 2; and five analyses between US$ 2 and US$ 5. The average cost per person treated was US$ 1.37 with a standard deviation (SD) of US$ 1.14.

Generally, a factor that might–at least partially–explain the variation in the costs was the inclusion of opportunity costs of personnel involved in service delivery and of transport costs. Fourteen of the cost analyses considered included opportunity costs, while 13 did not. The difference between financial and economic costs did not seem to play a role in the explanation of the costs’ variation. Overall, the cost estimates of more recent studies were considerably lower; yet, it was not possible to fully understand the potential reasons for this observed trend (Table 4).

**Table 4:** List of analyses and their unit costs ordered by year when the intervention occurred.

Reference	Group of studies per intervention	Total cost (US$) inflated to 2018	Costs for personnel and training	Cost for human drugs	Costs for materials and equipment	Costs for running the program, transport and management	Cost for diagnostic test (e.g., questionnaire)	Cost for behavior change	Other	Units of measurement used
Guyatt et al. (1994)	C	0.812	0.689	0.259	0.449	0.349	-	-	0.244	People treated
Guyatt et al. (1994)	C	0.837	0.879	0.259	0.454	0.360	-	-	0.822	People treated
Guyatt et al. (1994)	A	1.445	0.166	1.263	-	0.665	-	-	0.592	People treated
Guyatt et al. (1994)	A	1.450	0.173	1.219	-	0.668	-	-	0.142	People treated
Guyatt et al. (1994)	C	1.965	0.570	1.142	0.613	0.552	-	-	0.985	People treated
Guyatt et al. (1994)	C	2.069	0.163	1.219	0.645	0.579	-	-	0.232	People treated
Partnership for Child Development (1998)	B	1.195	0.131	0.876	-	0.786	0.875	-	0.278	Children treated
Partnership for Child Development (1999)	B	1.848	0.322	0.835	-	0.649	0.295	-	0.729	Children treated
Partnership for Child Development (1998)	B	1.996	0.131	0.876	-	0.574	0.388	-	0.278	Children treated
Partnership for Child Development (1999)	B	4.452	0.337	0.876	-	1.673	1.957	-	0.713	Children treated
Talaat & Evans (2000)	A	2.281	0.560	1.193	0.882	0.450	-	-	0.000	Children treated
Talaat & Evans (2000)	C	2.513	0.552	0.774	0.753	0.435	-	-	-	Children screened
Kabatereine et al. (2006)	A	0.058	0.123	0.513	-	0.486	-	0.457	-	People targeted
Oshish et al. (2011)	A	0.647	0.730	0.292	-	0.196	-	0.545	0.336	People targeted
Croce et al. (2010)	B	1.265	-	0.219	-	0.897	0.128	-	0.211	People protected/controlled
Guo et al. (2005)	C	0.349	0.571	0.233	0.582	-	-	-	0.000	People treated
Yu et al. (2002)	C	1.215	0.792	0.397	0.258	-	-	-	0.000	People surveyed
Guo et al. (2005)	A	0.827	0.653	0.762	-	-	-	-	-	People treated
Yu et al. (2002)	A	1.839	1.247	0.321	0.468	0.276	-	-	0.131	People surveyed
Yu et al. (2002)	C	1.320	0.776	0.528	0.168	-	-	-	0.697	People treated
Brooker et al. (2008)	A	0.689	0.116	0.272	-	0.179	-	0.122	-	People treated
Gabrielli et al. (2006)	A	0.408	0.555	0.283	0.737	0.512	-	0.155	-	Children treated
Leslie et al. (2011)	A	0.689	-	0.335	0.154	0.165	-	-	0.173	Treatments delivered
Linehan et al. (2011)	A	0.161	-	0.365	-	0.774	0.177	0.355	-	Treatments delivered
Evans et al. (2011)	A	0.054	0.472	-	0.372	0.275	-	-	-	Treatments delivered
Evans et al. (2011)	A	0.091	0.839	-	0.198	0.544	-	-	-	Treatments delivered
Leslie et al. (2013)	A	4.461	0.543	4.245	0.196	0.320	-	-	0.117	Treatments delivered

### Cost by type of intervention

Most of the cost estimates available pertained to preventive chemotherapy; 19 among 27 cost estimates. Among them, 9 exclusively focused on preventive chemotherapy, while 10 were combinations of preventive chemotherapy with other interventions and activities.

Group A included preventive chemotherapy studies with or without an educational component. The individual costs of the preventive chemotherapy intervention in group A ranged from a minimum value of US$ 0.06 per person treated to a maximum value of US$ 4.46 with an average of US$ 1.00. Excluding the study done by Leslie et al. (2013), which was an outlier as it estimated the costs of an integrated preventive chemotherapy intervention targeting several NTDs, the maximum value was US$ 2.28 and the average value was US$ 0.77.

Group B consisted of five studies, evaluating the costs of interventions to identify at-risk population. These costs varied from US$ 1.20 per person at-risk per year [37] to US$ 4.40 [25] for the intervention carried out in Ghana. Interestingly, the authors did the same intervention in Ghana and Tanzania and explained that the higher per-capita costs incurred in Ghana were due to the fact that in this country less people needed treatment. Hence, fixed costs were divided for a smaller number of people. The average financial cost of preventive chemotherapy plus an individual test to identify at-risk population was, on average, equal to US$ 2.15 per person, hence about twice as much as the average cost for group A.

In group C, test-and-treat interventions were included. These interventions were all preceded by a diagnostic test. The investigators of two articles employed the Kato-Katz thick smear method, three applied urine sedimentation, two used reagent strip tests, and one was based on the use of a simple questionnaire. Costs ranged from US$ 0.35 to US$ 2.50 with an average cost per person of US$ 1.45. The costs of the studies in group C were, on average, lower than those of the studies in group B, mainly due to lower costs for running the program.

### Cost categories for each cost analysis

One of the challenges of this cost analysis was to harmonize and compare the different cost categories across studies. In each study, costs were classified under different names and categories. Hence, we grouped them into main categories, similar for all the studies, namely: cost of personnel and training, cost for human drugs, cost for diagnostic tests (e.g., questionnaires, surveys, and urine centrifugation), costs for running the program (e.g., delivery of drugs, transport, management, coordination, planning, supervision, and drug distribution), costs for materials and equipment, costs for behavioral interventions (e.g. health education, sensitizations campaigns, and social mobilization).

Table 5 shows that, overall, the highest costs were those for drugs (49%), followed by the costs for personnel and training (21%), the costs related to running the program (18%), costs for materials and equipment (9%), costs for diagnostic tests (18%, where present) and, finally, costs for behavioral interventions (10%, where present) plus other not precisely identified costs (2%). In all groups, the highest cost component was represented by costs for human drugs. In groups A and C, the second biggest component was represented by cost of personnel, while in group B, the second highest component was represented by costs for running the program (35%) and the third component by costs for diagnostic tests (19%). Group C included also costs for materials and equipment (28%), beside cost of personnel (25%).

**Table 5:** Total unit costs and costs divided in categories.

Reference	Group of studies per intervention	Reference year(s) for intervention(s) and cost(s)	Total cost (US$) inflated to 2018	Costs for personnel and training	Cost for human drugs	Costs for materials and equipment	Costs for running the program, transport and management	Cost for diagnostic test (e.g., questionnaire)	Cost for behavior control	Other costs
Guo et al. (2005)	C	1998–2000	0.349	16%	67%	17%	-	-	-	0%
Partnership for Child Development (1998)	B	1996	1.195	11%	73%	-	7%	7%	-	2%
Yu et al. (2002)	C	1998–2002	1.839	65%	17%	3%	15%	-	-	0%
Guyatt et al. (1994)	C	1991	0.837	10%	31%	54%	4%	-	-	0%
Yu et al. (2002)	C	1998–2000	1.215	65%	33%	2%	-	-	-	0%
Partnership for Child Development (1999)	B	1996	1.848	17%	45%	-	35%	16%	-	4%
Partnership for Child Development (1998)	B	1996	1.996	7%	44%	-	29%	19%	-	1%
Brooker et al. (2008)	A	2003–2005	0.689	17%	40%	-	26%	-	18%	-
Gabrielli et al. (2006)	A	2004–2005	0.408	14%	69%	2%	13%	-	3%	0%
Guyatt et al. (1994)	A	1991	1.445	11%	83%	-	5%	-	-	0%
Kabatereine et al. (2006)	A	2003	0.058	2%	89%	-	8%	-	1%	0%
Linehan et al. (2011)	A	2006–2009	0.161	0%	19%	-	48%	11%	22%	-
Oshish et al. (2011)	A	2009	0.647	11%	45%	-	30%	-	8%	5%
Yu et al. (2002)	A	1998–2001	1.320	59%	40%	1%	-	-	-	0%
Guyatt et al. (1994)	C	1991	2.069	8%	58%	31%	3%	-	-	0%
Guyatt et al. (1994)	C	1991	1.965	3%	58%	31%	3%	-	-	5%
Guyatt et al. (1994)	C	1991	0.812	7%	31%	54%	4%	-	-	3%
Evans et al. (2011)	A	2008–2009	0.054	88%	-	7%	5%	-	-	0%
Evans et al. (2011)	A	2008–2009	0.091	92%	-	2%	6%	-	-	0%
Leslie et al. (2013)	A	2008–2009	4.461	1%	95%	0%	1%	-	-	2%
Leslie et al. (2011)	A	2004–2006	0.689	0%	49%	2%	24%	-	-	25%
Guyatt et al. (1994)	A	1991	1.450	12%	83%	-	5%	-	-	0%
Guo et al. (2005)	A	1998–2001	0.827	8%	92%	-	-	-	-	0%
Talaat & Evans (2000)	A	1999	2.281	25%	52%	4%	19%	-	-	0%
Croce et al. (2010)	B	1995–2006	1.265	0%	17%	-	71%	10%	-	2%
Talaat & Evans (2000)	C	1999	2.513	22%	31%	30%	17%	-	-	-
Partnership for Child Development (1999)	B	1996	4.452	8%	20%	-	36%	44%	-	2%

We found that some studies of group A, which pertained to preventive chemotherapy, also included some costs for behavioral interventions. In this category, costs varied considerably. The studies estimating low costs were those that estimated the costs of sensitizing activities more aimed at informing people on the preventive chemotherapy campaign, rather than real costs for the behavioral change intervention.

### Delivery costs

**Fig 2:**
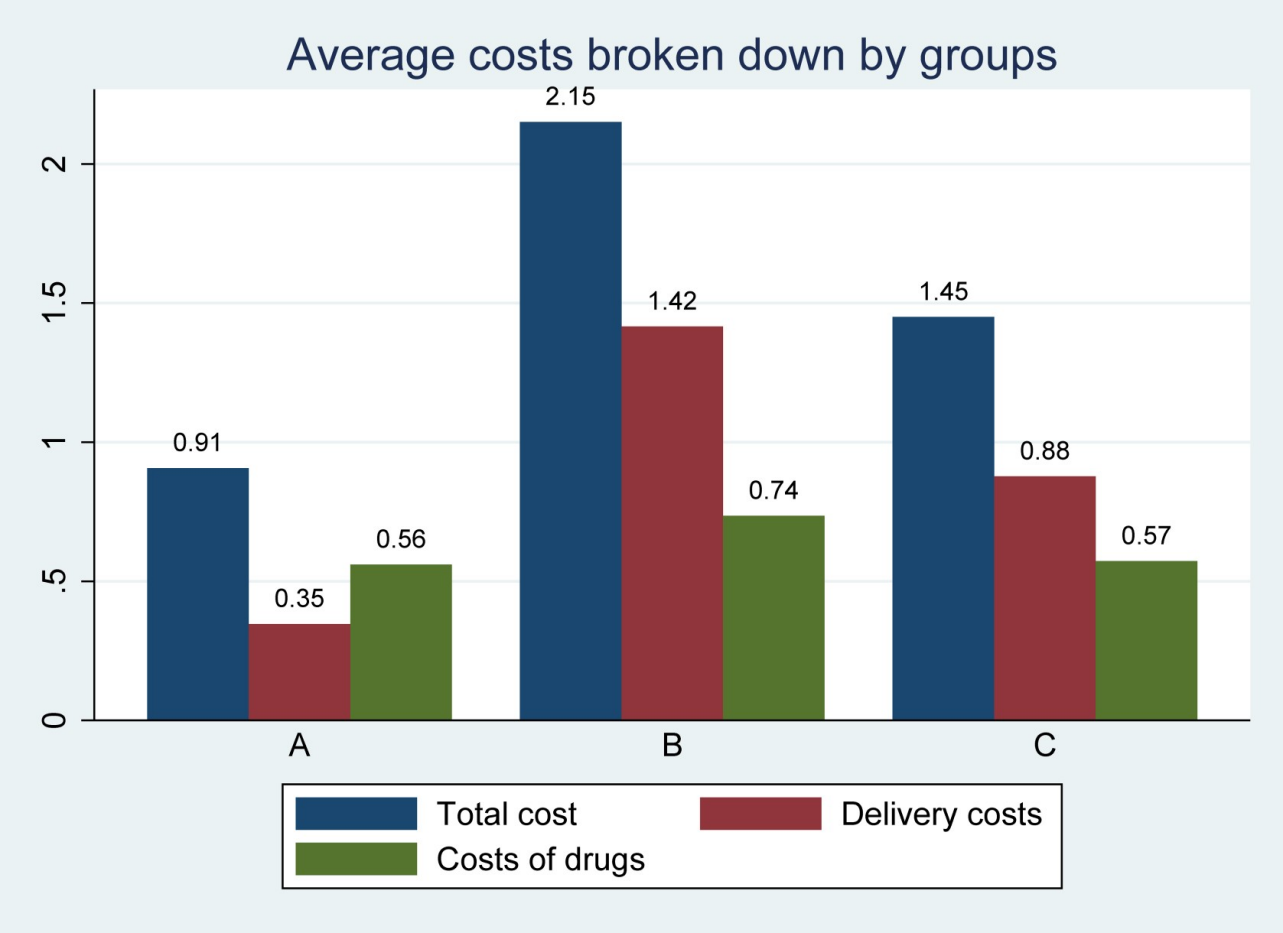
Total costs (US$) and costs divided in categories.

Fig 2 shows for each group the total costs, the drug, and the delivery costs. The latter are defined as total intervention costs minus the drug costs. The average delivery cost was US$ 0.35 for group A (US$ 0.30 if we include also the study of Leslie et al. (2013) [32] and that of Evans et al. (2011) [31]), US$ 1.42 for group B, and US$ 0.88 for group C. The higher costs for group C compared to group A were due to the fact that the intervention evaluated included patients treated with integrated care—generally more costly than patients treated during a vertical program. In contrast, the highest average cost of group B reflected the costs of a diagnostic test to select only infected people for subsequent treatment.

### Costs of the drug

In some of the preventive chemotherapy interventions considered in our review, drugs were donated by pharmaceutical companies [26, 32, 34, 37]. In these cases the drug price tried to reflect the market price of the country of the study [32] or it was taken as defined by the specific donation program [33]. Fig 2 shows the drug costs of each cost analyses for each group. The costs of drugs were very similar for groups A and C (costs per treatment were US$ 0.56 and US$ 0.57, respectively), while the costs were slightly higher for group B (US$ 0.74).

Note: The paper by Leslie et al. (2013) and the paper by Evans et al. (2011) are excluded from this graph because the former has very high costs and the latter has zero costs imputed.

### Economic costs

About half of the cost estimates considered included the opportunity costs of the personnel involved in delivering the intervention, although this activity did not generate additional financial compensation. Economic personnel costs were generally converted into monetary values based on the average daily income of each professional category. These costs included, in particular, the value of the time spent by the laboratory technicians to perform the Kato-Katz thick smear test, the time spent by nurses to prescribe the medicine and treat the patients, opportunity cost of employing existing Ministry of Health staff and school teachers [23, 33], time of volunteer distributers [32], or more generally non-health professionals who spent their productive time on activities that did not involve additional financial compensation or any form of gifts [24, 25]. In a few studies, the capital costs were also included in the economic costs. In some studies, capital costs were annualized and the equivalent annual cost represented value-in-use of capital items [23]. Other studies stated to account for the time a vehicle was used [33], or more generally also the unpaid transport costs among the economic costs considered [24, 25]. One study mentioned the opportunity costs of purchasing of height poles–used to calculate the dose of praziquantel to treat urogenital schistosomiasis on the basis of height of the child [40]–and the installation of security doors for the safe storage of drugs [26]. Other studies generally stated to consider economic costs but did not explicitly explain how they consider capital costs in the analyses [25, 28, 29, 32, 33].

On average, the delivery costs were more than two times higher in the analyses which included economic costs (US$ 1.02 *versus* US$ 0.44), as expected. Yet there is variation in the type of opportunity costs evaluated by the studies. For instance, in one study [28], the authors considered in their opportunity costs only the time of skilled staff, which only had a minimal effect on the total economic costs (+3%). A bigger weight of the economic costs was computed in other studies such as [24] and [25], where they were 67% and 140% higher than the financial costs, respectively, and included also opportunity costs related to transport and other activities.

## Discussion

Estimating the costs of schistosomiasis control and elimination interventions is important to inform decisions on affordability of programs, on how to allocate resources, and as input for broader economic analyses. Our results indicated that for preventive chemotherapy interventions, costs ranged from US$ 0.05 [31] to US$ 4.46 [32] per person treated, for preventive chemotherapy plus an individual diagnostic test to identify at-risk populations, costs ranged from US$ 1.19 [24] to US$ 4.45 [25] per person treated, while for test-and-treat interventions, costs ranged from US$ 0.35 [27] to US$ 2.51 [29] per person treated.

The review presented here highlights that, although the literature on schistosomiasis control interventions costs is vast, only a small number of studies contained a level of detail that allowed analyzing cost estimates. The studies identified used quite different approaches to estimate the costs of the intervention assessed. For example, several studies based their cost assessments on program budgets/reports and only very few were based on comprehensive costing studies/approaches. Hence, most of the studies initially retrieved by our systematic search could not be analyzed. Among the 15 studies finally included in our cost analysis, there was considerable variation in the types of costs. Moreover, it was hard to compare the reported costs across studies due to several reasons, including lack of clarity regarding how the costs were estimated, and which activities were included in the final costing.

Our study has several limitations. First, our search strategy considered only electronic databases and sources. Due to the high number of studies retrieved, the selection process required some simplified procedures in the first selection steps that may represent a potential source of bias, similar to other reviews of costing studies [41]. Second, the studies included in the review revealed that, despite a certain degree of standardization of the schistosomiasis interventions, each study analyzed presented several specificities that made comparability very hard. Interestingly the cost estimates reviewed were not systematically correlated to specific aspects that should influence the costs, such as the country where the study was conducted or the number of people treated. The study that estimated costs of programs that were targeting not only schistosomiasis but also other NTDs were particularly difficult to interpret as attributing the costs to schistosomiasis only was problematic. Third, similarly to what was found in other reviews [41], it was not possible to draw firm conclusions on the costs of the different interventions. This was due to variation and inconsistencies in the analytical methods adopted and a general lack of details provided.

Our review highlights the limitations and challenges of the existing literature on the costs of schistosomiasis interventions. Most of these challenges are similar to those reported in the literature not only of other NTDs but also of global health interventions more generally. A couple of lessons can be learned from our review. First, conducting rigorous costing studies is a time consuming and complex task that requires striking a balance between comparability of results, external validity, and taking into account the complexity of the context in which specific interventions were deployed. Although there are several methodological guidelines on how to conduct and report economic evaluations of health interventions, there is still wide variation in the methods used and in the quality of the cost estimates and in how they are reported. As shown by the lack of relation between the declared perspective of the studies and the types of costs included, the level of uptake of key principles of economic evaluations in the health sectors is still low. This indicates that the NTD communities should invest more in developing economic evaluations skills. Clearly, there is a need for investments in research conducted by multidisciplinary research teams, including both NTD experts and health economists. Second, clear and transparent reporting of the results of economic evaluation and costing studies is crucial for their usefulness to inform policy decisions and guiding resource allocation. Yet, the degree of transparency of most of the costing studies pertaining to schistosomiasis interventions identified in our review was limited. All costing studies should at least transparently present the methods used to identify the resources used by an intervention, how these resources were measured and quantified, and how they were given a financial or economic value. Ideally, they should also present the breakdown of the costs by at least the macro cost components by activities and input. Lastly, the population reached by the intervention evaluated, should always be explicitly mentioned. Hence, there is a pressing need for higher reporting standards and transparency that should be fostered by peer-review journal policies. The practice of submitting web appendixes and datasets could probably address this challenge although it would require substantial documentation efforts.

Despite the limitations of this review and the methodological challenges of the studies included, the results provide some policy relevant indications. First, the costs of schistosomiasis interventions are relatively low compared to those of other global health interventions. Yet, the drug delivery costs that are usually borne by endemic countries, may still not be affordable for many low income countries in particular if the economic costs are considered. This triggers the questions of how and when interventions can be sustainably integrated into existing health systems and fully owned, administered, and paid for by the countries themselves. Donor support might need to continue for the next years to guarantee that the success made in schistosomiasis control and elimination to date is sustained, and that there is no bounce back of infections and diseases in case countries cannot afford the interventions. Second, the cost estimates included in this review could be used by policy makers and NTD program managers for estimating the affordability of their programs and to assess the importance of the different cost components. Lastly, they could also be used as input for other studies aiming to assess the cost-effectiveness of different schistosomiasis control or elimination interventions.

## Supporting information

S1 Table List of costs analyses of group A and their main characteristics. (DOCX)

S2 Table List of costs analyses of groups B and C and their main characteristics. (DOCX)

S3 Table Summary of the search strategy and retrieved number of raw hits. (DOCX)

S4 Table Details on the search strategy development and rationale. (DOCX)

S5 Table PRISMA 2009 checklist. (DOC)
