# Supplementary material for: Cost of interventions to control schistosomiasis: A systematic review of the literature
Source: PLoS Negl Trop Dis. 2020 Mar 30;14(3):e0008098. doi: 10.1371/journal.pntd.0008098 (PMC7145200; doi:10.1371/journal.pntd.0008098)
Supplement: S1 Table — (DOCX) [file pntd.0008098.s001.docx]

**Table S1: List of costs analyses of group A (i.e. preventive chemotherapy with or without an educational component) and their main characteristics**

| **Reference** | **Group of studies per intervention** | **Country** | **Reference year(s) for intervention(s) and cost(s)** | **Target of intervention** | **Type of economic analysis** | **Economic perspective explicitly stated** | **Economic costs included (Y/N)** | **Diagnostic test** | **MDA (Y/N)** | **Individual human treatment (Y/N)** | **ICE or BC (Y/N)** | **Units of measurement used** | **How many?** |
| --- | --- | --- | --- | --- | --- | --- | --- | --- | --- | --- | --- | --- | --- |
| Brooker et al. (2008) | A | UGANDA | 2003-2005 | Schisto and STH | CEA | Government | Y | None | Y | N | Y | People treated | 408352 |
| Evans et al. (2011) | A | NIGERIA | 2008-2009 | Schisto, Onchocerciasis, LF and STH | CEA | NA | N | None | Y | N | N | Treatments delivered | 1581369 |
| Evans et al. (2011) | A | NIGERIA | 2008-2009 | Schisto, Onchocerciasis, LF and STH | CEA | NA | N | None | Y | N | N | Treatments delivered | 1596587 |
| Gabrielli et al. (2006) | A | BURKINA FASO | 2004-2005 | Schisto | Costing | NA | Y | None | Y | N | Y | Children treated | 3322564 |
| Guo et al. (2005) | A | CHINA | 1998-2001 | Schisto | Costing | NA | N | None | Y | N | N | People treated | 724 |
| Guyatt et al. (1994) | A | TANZANIA | 1991 | Schisto | CEA | Health care's provider | N | None | Y | N | N | People treated | 18094 |
| Guyatt et al. (1994) | A | TANZANIA | 1991 | Schisto | CEA | Health care's provider | Y | None | Y | N | N | People treated | 18094 |
| Kabatereine et al. (2006) | A | UGANDA | 2003 | Schisto and and STH | Costing | NA | N | None | Y | N | N | People targeted | 546730 |
| Leslie et al. (2011) | A | NIGER | 2004-2006 | Schisto and and STH | CEA | NA | Y | None | Y | N | Y | Treatments delivered | 818562 |
| Leslie et al. (2013) | A | NIGER | 2008-2009 | LF, schisto, trachoma and STH | Costing | NA | Y | None | Y | N | Y | Treatments delivered | 5198031 |
| Linehan et al. (2011) | A | BURKINA FASO, GHANA, MALI, NIGER, UGANDA, SIERRA LEONE, HAITI (only LF and STH in HAITI) | 2006-2009 | Schisto, LF, oncho, STH, trachoma | Costing | NA | N | Parasitologic methods for schisto: 1) eggs in urine or stool (microscopy)  2) blood in urine (hemastix or questionnaires) | Y | N | Y | Treatments delivered | 222700176 |
| Oshish et al. (2011) | A | YEMEN | 2009 | Schisto & STH | Costing | NA | N | None | Y | N | Y | People targeted | 829242 |
| Talaat & Evans (2000) | A | EGYPT | 1999 | Schisto | CEA | NA | Y | None | Y | N | N | Children treated | 30440 |
| Yu et al. (2002) | A | CHINA | 1998-2001 | Schisto | CEA | Health care's provider | N | None | Y | N | N | People treated | 1727 |

Notes: In column “Target of intervention” STH stands for soil-transmitted helminths, LF for lymphatic filariasis and “Schisto” for schistosomiasis. In column “Type of economic analysis” CEA indicates a cost-effectiveness analysis. The column MDA indicates whether the intervention was a Mass Drug Administration or not. IEC or BC indicates whether the main intervention included also a specific Information, Education, and Communication campaign or a Behavioural Control intervention.
