# Supplementary material for: Cost of interventions to control schistosomiasis: A systematic review of the literature
Source: PLoS Negl Trop Dis. 2020 Mar 30;14(3):e0008098. doi: 10.1371/journal.pntd.0008098 (PMC7145200; doi:10.1371/journal.pntd.0008098)
Supplement: S2 Table — (DOCX) [file pntd.0008098.s002.docx]

**Table S2: List of costs analyses of group B (i.e. preventive chemotherapy plus an individual test to identify at-risk population) and group C (test-and-treat interventions) and their main characteristics.**

| **Reference** | **Group of studies per intervention** | **Country** | **Reference year(s) for intervention (s) and cost(s)** | **Target of intervention** | **Type of economic analysis** | **Economic perspective explicitly stated** | **Economic costs included (Y/N)** | **Diagnostic test** | **MDA (Y/N)** | **Individual human treatment (Y/N)** | **ICE or BC (Y/N)** | **Units of measurement used** | **How many?** |
| --- | --- | --- | --- | --- | --- | --- | --- | --- | --- | --- | --- | --- | --- |
| Croce et al. (2010) | B | CAMBODIA | 1995-2006 | Schisto | CEA | Ministry of Health | Y | An average of 2000 stool samples from individuals in randomly selected villages were collected for parasitological survey and analyzed with the Kato-Katz method. | Y | N | (Y/N) | People protected | 71364 |
| Partnership for Child Development (1998) Health Policy Plan | B | TANZANIA | 1996 | Schisto | Costing | NA | Y | Questionnaire to decide which schools to treat. | Y | N | N | Children treated | 39372 |
| Partnership for Child Development (1998) | B | TANZANIA | 1996 | Schisto | Costing | NA | Y | Questionnaire to decide which schools to treat. | Y | N | N | Children treated | 39372 |
| Partnership for Child Development (1999) | B | GHANA | 1996 | Schisto | Costing | NA | Y | Questionnaire to pupils to self-assess if they had blood in urine. | Y | N | N | Children treated | 15325 |
| Partnership for Child Development (1999) | B | GHANA | 1996 | Schisto | Costing | NA | Y | Questionnaire to pupils to self-assess if they had blood in urine. | Y | N | N | Children treated | 15325 |
| Guo et al. (2005) | C | CHINA | 1998-2000 | Schisto | Costing | NA | N | Kato- Katz method. | N | Y | Y | People treated | 846 |
| Guyatt et al. (1994) | C | TANZANIA | 1991 | Schisto | CEA | Health care's provider | N | Reagent strip tests. | N | Y | N | People treated | 18094 |
| Guyatt et al. (1994) | C | TANZANIA | 1991 | Schisto | CEA | Health care's provider | Y | Reagent strip tests. | N | Y | N | People treated | 18094 |
| Guyatt et al. (1994) | C | TANZANIA | 1991 | Schisto | CEA | Health care's provider | N | Passive case detecting using urine sedimentation. | N | Y | N | People treated | 29920 |
| Guyatt et al. (1994) | C | TANZANIA | 1991 | Schisto | CEA | Health care's provider | Y | Passive case detecting using urine sedimentation. | N | Y | N | People treated | 29920 |
| Talaat & Evans (2000) | C | EGYPT | 1999 | Schisto | CEA | NA | Y | Screening involved only urine using the simple sedimentation technique. | N | Y | N | Children screened | 30440 |
| Yu et al. (2002) | C | CHINA | 1998-2000 | Schisto | CEA | Health care's provider | N | Simple questionnaire interview of the individuals inquiring about the history related to schistosome infection. | N | Y | N | People surveyed | 1691 |
| Yu et al. (2002) | C | CHINA | 1998-2002 | Schisto | CEA | Health care's provider | N | Kato- Katz method. | N | Y | N | People surveyed | 1039 |

Notes: In column “Target of intervention” “Schisto” stands for schistosomiasis. In column “Type of economic analysis” CEA indicates a cost-effectiveness analysis. The column MDA indicates whether the intervention was a Mass Drug Administration or not. IEC or BC indicates whether the main intervention included also a specific Information, Education, and Communication campaign or a Behavioural Control intervention.
