# Supplementary material for: Cost of interventions to control schistosomiasis: A systematic review of the literature
Source: PLoS Negl Trop Dis. 2020 Mar 30;14(3):e0008098. doi: 10.1371/journal.pntd.0008098 (PMC7145200; doi:10.1371/journal.pntd.0008098)
Supplement: S3 Table — (DOCX) [file pntd.0008098.s003.docx]

**TableS3: Summary of the search strategy and retrieved number of raw hits**

| **Database** | **Thesaurus** | **Search term** | **Number of hits** |
| --- | --- | --- | --- |
|  |  |  | **(30.04.2019)** |
| PubMed | MeSH | (Schistosomiasis [MeSH] OR Schistosoma [MeSH]) AND (Economics [MeSH] OR Program Evaluation [MeSH]) | 273 |
| WHOLIS | MeSH | (Schistosomiasis [MeSH] OR Schistosoma [MeSH]) AND (Economics [MeSH] OR Program Evaluation [MeSH]) | 6 |
| ISI Web of Science [All Databases] | No thesaurus; basic keyword search | (Schistosom*[topic]) AND (Econom*[topic] OR Cost*[topic]) | 3485 |
