# Supplementary material for: Cost of interventions to control schistosomiasis: A systematic review of the literature
Source: PLoS Negl Trop Dis. 2020 Mar 30;14(3):e0008098. doi: 10.1371/journal.pntd.0008098 (PMC7145200; doi:10.1371/journal.pntd.0008098)
Supplement: S4 Table — (DOCX) [file pntd.0008098.s004.docx]

**Table S4: Details on the search strategy development and rationale.**

| **Database** | **Thesaurus** | **Search term** | **Boolean** | **Number of hits** |
| --- | --- | --- | --- | --- |
|  |  |  | **operator** | **(30.04.2019)** |
| PubMed | MeSH | Schistosomiasis | OR | 194 |
|  | MeSH | Schistosoma | AND |  |
|  | MeSH | Economics |  |  |
| PubMed | MeSH | Schistosomiasis | OR | 57 |
|  | MeSH | Schistosoma | AND |  |
|  | MeSH | Program Evaluation |  |  |
| PubMed | MeSH | Schistosomiasis | OR | 3,188 |
|  | MeSH | Schistosoma | AND |  |
|  | MeSH subheading | Prevention and control |  |  |
| PubMed | MeSH | Schistosomiasis | OR | 273 |
|  | MeSH | Schistosoma | AND |  |
|  | MeSH | Economics | OR |  |
|  | MeSH | Program Evaluation |  |  |
| WHOLIS | MeSH | Schistosomiasis | AND | 2 |
|  | MeSH | Economics |  |  |
| WHOLIS | MeSH | Schistosoma | AND | 0 |
|  | MeSH | Economics |  |  |
| WHOLIS | MeSH | Schistosomiasis | AND | 4 |
|  | MeSH | Program Evaluation |  |  |
| WHOLIS | MeSH | Schistosoma | AND | 0 |
|  | MeSH | Program Evaluation |  |  |
| WHOLIS | MeSH | Schistosomiasis | AND | 161 |
|  | MeSH subheading | Prevention and control |  |  |
| WHOLIS | MeSH | Schistosoma | AND | 1 |
|  | MeSH subheading | Prevention and control |  |  |
| WHOLIS | MeSH | Schistosomiasis | OR | 6 |
|  | MeSH | Schistosoma | AND |  |
|  | MeSH | Economics | OR |  |
|  | MeSH | Program Evaluation | OR |  |
| ISI Web of Science | no thesaurus; | Schistosom* |  | 69645 |
| [All Databases] | basic keyword |  |  |  |
|  | search |  |  |  |
| ISI Web of Science | no thesaurus; | Econom* |  | 4,241,902 |
| [All Databases] | basic keyword |  |  |  |
|  | search |  |  |  |
| ISI Web of Science | no thesaurus; | Cost* |  | 6,322,878 |
| [All Databases] | basic keyword |  |  |  |
|  | search |  |  |  |
| ISI Web of Science | no thesaurus; | Schistosom* | AND | 2307 |
| [All Databases] | basic keyword | Econom* |  |  |
|  | search |  |  |  |
| ISI Web of Science | no thesaurus; | Schistosom* | AND | 1697 |
| [All Databases] | basic keyword | Cost* |  |  |
|  | search |  |  |  |
| ISI Web of Science | no thesaurus; | Schistosom* | AND | 3485 |
| [All Databases] | basic keyword | Econom* | OR |  |
|  | search | Cost* |  |  |

Note: This table shows the details of the of the search strategy results summarized in Table S2. the last row of Table S3 (ISI Web of science) is 3485 that is equal to the number reported in Table S2 last row; the number of WHOLIS in Table S3 last row of WHOLIS is 6 that is also reported in Table S2 second row; the number of Pubmed in Table S3 last row of Pubmed is 273 that is equal to the number reported in Table S2 first row (Pubmed).
